# Icariside II, a Naturally Occurring SIRT3 Agonist, Protects against Myocardial Infarction through the AMPK/PGC-1α/Apoptosis Signaling Pathway

**DOI:** 10.3390/antiox11081465

**Published:** 2022-07-27

**Authors:** Yeli Li, Linying Feng, Dianyou Xie, Mu Lin, Yiqi Li, Nana Chen, Danli Yang, Jianmei Gao, Yizhun Zhu, Qihai Gong

**Affiliations:** 1 Department of Pharmacy, Macau University of Science and Technology, Macau 999078, China; liyeli@zmu.edu.cn (Y.L.); 2009853qpp30001@student.must.edu.mo (L.F.); 2109853qpp30003@student.must.edu.mo (D.X.); 2109853qpp30002@student.must.edu.mo (M.L.); gaojianmei@zmu.edu.cn (J.G.); yzzhu@must.edu.mo (Y.Z.); 2 Key Laboratory of Basic Pharmacology of Ministry of Education and Joint International Research Laboratory of Ethnomedicine of Ministry of Education, Zunyi Medical University, Zunyi 563000, China; lyq@zmu.edu.cn (Y.L.); cnn@zmu.edu.cn (N.C.); yangdanli@zmu.edu.cn (D.Y.); 3 Department of Clinical Pharmacotherapeutics, School of Pharmacy, Zunyi Medical University, Zunyi 563000, China

**Keywords:** Icariside II, myocardial infarction, oxidative stress, SIRT3, AMPK/PGC-1α/apoptosis pathway

## Abstract

Myocardial infarction (MI) refers to the death of cardiomyocytes triggered by a lack of energy due to myocardial ischemia and hypoxia, and silent mating type information regulation 2 homolog 3 (SIRT3) plays an essential role in protecting against myocardial oxidative stress and apoptosis, which are deemed to be the principal causes of MI. Icariside II (ICS II), one of the main active ingredients of *Herbal Epimedii*, possesses extensive pharmacological activities. However, whether ICS II can protect against MI is still unknown. Therefore, this study was designed to investigate the effect and possible underlying mechanism of ICS II on MI both in vivo and in vitro. The results showed that pretreatment with ICS II not only dramatically mitigated MI-induced myocardial damage in mice but also alleviated H9c2 cardiomyocyte injury elicited by oxygen and glucose deprivation (OGD), which were achieved by suppressing mitochondrial oxidative stress and apoptosis. Furthermore, ICS II elevated the phosphorylation level of adenosine monophosphate-activated protein kinase (AMPK) and peroxisome proliferator-activated receptor-gamma coactivator 1 alpha (PGC-1α) expression, thereby activating SIRT3. However, these protective effects of ICS II on MI injury were largely abolished in SIRT3-deficient mice, manifesting that ICS II-mediated cardioprotective effects are, at least partly, due to the presence of SIRT3. Most interestingly, ICS II directly bound with SIRT3, as reflected by molecular docking, which indicated that SIRT3 might be a promising therapeutic target for ICS II-elicited cardioprotection in MI. In conclusion, our findings illustrate that ICS II protects against MI-induced oxidative injury and apoptosis by targeting SIRT3 through regulating the AMPK/PGC-1α pathway.

## 1. Introduction

Myocardial infarction (MI) is the most common ischemic heart disease and a leading cause of morbidity and mortality worldwide, which is characterized by the obstruction of blood flow in coronary arteries, thereby depriving cardiomyocytes of oxygen and nutrients and leading to the demise of cardiomyocytes [1,2]. Unfortunately, ideal therapies to treat MI are unavailable. Currently, the applications of percutaneous coronary intervention, coronary artery bypass grafting, and thrombolytic treatment are limited in clinics due to their poor prognosis and serious complications such as hemorrhage and myocardial ischemia–reperfusion injury [3,4]. Encouragingly, most of those who are vulnerable to or at risk of MI, as well as a sharp increase in morbidity or mortality in the identified high-risk groups, can be forecasted [5]; thus, prophylactic treatment provides an alternative ideal strategy for the occurrence of MI.

At present, it is generally recognized that mitochondrial oxidative stress and apoptosis are involved in the pathological mechanisms of MI [6,7,8]. Oxidative stress is considered an imbalance between reactive oxygen species (ROS) generation and antioxidant defense, and the suppression of oxidative stress is conducive to alleviating MI [9,10]. Furthermore, apoptosis, an important downstream mediator of oxidative stress, plays an essential role during MI [11,12,13]. Notably, previous studies indicate that the adenosine-monophosphate-activated protein kinase (AMPK)/silent mating type information regulation 2 homolog 3 (SIRT3) pathway plays a critical role in regulating mitochondrial oxidative stress [8,14]. AMPK, the key energy sensor, is a pivotal regulator of myocardial ischemia and ensures the protection of MI via alleviating myocardial oxidative stress and apoptosis [15]. The activation of AMPK reduces mitochondrial ROS production by enhancing the expression of peroxisome proliferator-activated receptor-gamma coactivator 1 alpha (PGC-1α) [16]. Furthermore, PGC-1α also acts as an upstream regulator to regulate the expression of SIRT3, which is an important guardian of mitochondrial homeostasis, thereby regulating oxidative stress and apoptosis [17]. Since the downregulation of the AMPK/PGC-1α/SIRT3 pathway evokes mitochondrial dysfunction and aggravates myocardial injury after MI, the activation of the AMPK/PGC-1α/SIRT3 pathway can be an effective strategy to protect against myocardial oxidative stress and apoptosis after MI.

Icariside II (ICS II), a major active ingredient of the traditional Chinese medicine *Herbal Epimedii*, has extensive pharmacological activities such as antioxidant, anti-inflammation, and antitumor properties [18,19,20,21]. Our previous studies demonstrated that ICS II attenuated oxygen and glucose deprivation (OGD)-induced PC12 cell oxidative injury by activating nuclear factor erythroid 2-related factor 2/SIRT3 signaling pathway and alleviated left ventricular remodeling and apoptosis in spontaneously hypertensive rats [22,23]. In addition, recent studies have reported that ICS II attenuates myocardial ischemia and reperfusion injury by activating the PI3K/Akt signaling pathway [24]. However, ICS II-mediated cardioprotection and its potential therapeutic target in permanent ischemia remain unknown. Therefore, the current study was designed to explore the detailed mechanism of ICS II against left anterior descending coronary artery ligation-induced MI in wild-type (WT) and SITR3-deficient (SIRT3-KO) mice in vivo and OGD-induced injury in cardiomyocytes in vitro.

## 2. Materials and Methods

### 2.1. Animals

Male WT C57BL/6J mice (8–10 weeks) were supplied by the Hunan SJA Laboratory Animal Co., Ltd. (Hunan, China; Certificate No. SCXK 2019-0004). Heterozygous mice (presence of SIRT3; SIRT3^−/+^) were purchased from Cyagen Biosciences Inc. (Certificate No. SYXK (Su)-2018-0005, Suzhou, China). SIRT3-null mice (absence of SIRT3; SIRT3-KO) were purified and bred from heterozygous mice. All mice were raised with a 12 h light/dark cycle and provided free access to standard rodent diet and water and kept under humidity (55  ±  5%) and temperature (23  ±  1 °C) controlled environment. Randomization was used to allocate animals to different experimental groups, and the data analysis was performed by a blinded investigator. All animal experimental procedures in the present study were sanctioned by the Experimental Animal Ethics Committee of the Zunyi Medical University (Guizhou, China, No. ZMU21-2203-538) and conformed to the Guide for the Care and Use of Laboratory Animals published by the US National Institutes of Health (National Institutes of Health Publication 85–23, revised 1996).

### 2.2. MI Model and Drug Administration

Mice were treated by intragastric administration with ICS II (purity ≥ 98%, Nanjing Zelang Medical Technology Corporation Ltd., Nanjing, China) at different doses of 5, 10, and 20 mg/kg twice a day for 7 days before the subsequent experiments. The left anterior descending coronary artery was ligated to induce the MI model as described previously [25]. Briefly, mice were anesthetized with 1.5% isoflurane. Thereafter, the heart was manually exposed through a left thoracotomy between the third and fourth intercostal space, and a 6-0 silk suture was then looped around the left anterior descending coronary artery. Sham-operated mice underwent an identical procedure except that the suture was passed around the vessel without left anterior descending coronary artery occlusion.

Protocol 1: To investigate the effect of ICS II on MI, the mice were randomly divided into six groups: sham group, ICS II (20 mg/kg) + sham group, MI group, ICS II (5 mg/kg) + MI group, ICS II (10 mg/kg) + MI group, and ICS II (20 mg/kg) + MI group. Protocol 2: To confirm the role of SIRT3 in the anti-MI effect of ICS II, the mice were randomly divided into six groups: WT-sham group, WT-MI group, ICS II (20 mg/kg) + WT-MI group, SIRT3-KO-sham group, SIRT3-KO-MI group, and ICS II (20 mg/kg) + SIRT3-KO-MI group. The mice were administrated with ICS II twice a day for 7 days prior to MI, and the mice of sham and MI groups received volume-matched normal saline, instead.

### 2.3. Determination of Echocardiography

Briefly, 48 h after MI, the mice were anesthetized with 1.5% isoflurane; then, the B- and M-mode images of the left ventricle were obtained using a Vevo2100 imaging system (VisualSonics Corporation, Toronto, ON, Canada). The left ventricular ejection fraction (EF) and fractional shortening (FS) were then calculated from M-mode recording.

### 2.4. Assessment of Myocardial Infarct Size

After cardiac function estimation, the blood was collected, and then the heart was harvested and frozen at −20 °C for 30 min, sectioned into 2 mm thick transverse slices, and incubated in 0.5% 2,3,5-triphenyl tetrazolium chloride (TTC, Sigma-Aldrich, St. Louis, MO, USA) at 37 °C for 15 min. The heart slices were photographed, and digital images were quantified via planimetry using ImageJ. Infarct size (%) was calculated as the percent of myocardial infarct size/left ventricular size for any heart.

### 2.5. Microarray Processing and Data Analysis

The total RNAs were extracted from the heart tissues of mice in Sham, MI, and ICS II (20 mg/kg) + MI groups using a Trizol buffer and enriched and purified using oligo dT beads; then, RNA fragments and first-strand cDNAs were generated using random N6-primed reverse transcription, followed by second-strand cDNA synthesis with dUTP instead of dTTP. Next, the synthesized cDNA was subjected to end-repair and then was 3′-adenylated. Adaptors were ligated to the ends of these 3′-adenylated cDNA fragments, and PCR amplification, single-strand separation and cyclization, and DNA nanoball synthesis and sequencing on a DNBSEQ (DNBSEQ Technology) platform were then performed. The gene expression was calculated using the FPKM value of each sample; gene expression with a fold change (FC) greater than 2.0 and a *p*-value less than 0.05 were identified as differentially expressed genes (DEGs) in this study. Then, the significant enrichment analysis of Gene Ontology (GO) function on DEGs among the samples, the significant enrichment analysis of pathway and clustering, and more in-depth mining analysis were performed.

### 2.6. Hematoxylin–Eosin (HE) Staining

The heart specimens were fixed in 4% formaldehyde for 24 h, enclosed in paraffin, stained with HE according to the standard protocols, and observed by an optical microscope (Olympus BX43, Tokyo, Japan).

### 2.7. Transmission Electron Microscopy (TEM)

The left ventricular tissues were fixed with 2.5% glutaraldehyde in 0.1 M phosphate-buffered saline (pH 7.4) and then post-fixed in 1% osmium tetroxide for 2 h. After the samples were dehydrated in graded ethanol and embedded in epoxy resin, ultrathin sections were prepared and counterstained with uranyl acetate and lead citrate and then were observed under a transmission electron microscope (JEM-1400Flash, JEOL, Tokyo, Japan).

### 2.8. TdT-Mediated dUTP Nick-End Labeling (TUNEL) Staining

Myocardial apoptosis was evaluated via TUNEL staining using a one-step TUNEL apoptosis assay kit according to the manufacturer’s protocols. In short, the heart specimens were stained with TUNEL at 37 °C for 1 h, then stained with DAPI at room temperature for 8 min, and thereafter examined with a fluorescence microscope (Olympus BX53, Tokyo, Japan). Green fluorescein represents apoptotic nuclei, and the percentage of apoptotic nuclei (%) was calculated by the percent of TUNEL-positive nuclei (green)/total nuclei (blue).

### 2.9. Cell Culture and OGD Model

H9c2 cells (secondary generation cells, ATCC, Manassas, VA, USA) were cultured in Dulbecco’s modified Eagle’s medium (DMEM) containing 4 mM L glutamine, 4.5 g/L glucose, 10% fetal bovine serum (FBS), and antibiotics (100 U/mL penicillin and 100 μg/mL streptomycin) and were incubated in a humidified chamber (37 °C, 5% CO_2_). OGD was performed to emulate the MI model in vitro. In brief, 1 h before OGD, the cells were pretreated with different concentrations of ICS II (2, 4, and 8 μM). Cells were then incubated at 37 °C for 4 h in glucose-free and FBS-free DMEM in an oxygen-free N_2_/CO_2_ (95%/5%) environment. Thereafter, the cells were replaced with standard culture media or treated with different concentrations of ICS II and subjected to normoxic conditions for another 24 h.

### 2.10. Determination of Cell Viability and Cytotoxicity

Cardiomyocytes were treated as described above. Briefly, at the end-point of the treatment, 3-(4,5-dimethythiazol-2-yl)-2,5-diphenyltetrazolium bromide (MTT, 5 mg/mL) was added to each well and cultured for another 4 h. The medium was then discarded and the formazan dissolved using DMSO. Thereafter, the absorbance of formazan formation was evaluated at 490 nm wavelength using a microplate reader. In addition, cytotoxicity was detected using a lactate dehydrogenase (LDH) kit as described previously [26]. In brief, after cardiomyocytes were treated as mentioned above, the supernatants were acquired and centrifuged at 825× *g* for 20 min at 4 °C. Furthermore, cellular morphologic changes were examined using a reverse-phase microscope.

### 2.11. Determination of LDH, Troponin (Tn), and TnT Levels

The cultured supernatants and animal serums were collected and centrifuged at 825× *g* for 20 min at 4 °C. Thereafter, Tn and TnT levels in the cultured supernatants and LDH, Tn, and TnT levels in the animal serums were detected using related ELISA kits according to the manufacturer’s protocols.

### 2.12. Measurement of ROS Generation

The intracellular ROS and mitochondrial superoxide anions (O_2_^•−^) were measured via dihydroethidium (DHE) and Mito-SOX red staining. In brief, after cardiomyocytes were treated as mentioned above and stained with 10 µM DHE at 37 °C for 30 min or 5 µM Mito-SOX Red at 37 °C for 10 min in the darkroom, they were immersed into DAPI at room temperature for 8 min. Images were captured using a fluorescence microscope (Olympus BX53, Tokyo, Japan). Fluorescence intensity was quantified using ImageJ.

### 2.13. Determination of Oxidative Stress-Related Indicators

The cultured supernatants and animal serums were collected and centrifuged at 825× *g* for 20 min at 4 °C. Thereafter, the levels of ROS and malondialdehyde (MDA), and glutathione peroxidase (GSH-Px), catalase (CAT), superoxide dismutase (SOD), SOD1, SOD2, and SOD3 activities were detected using related ELISA kits following the manufacturer’s procedures.

### 2.14. Flow Cytometric Analysis

Cardiomyocytes were treated as described above. In short, at the end of treatment, the cultured supernatants were collected, and the cells were digested with 0.25% EDTA-free trypsin and washed with PBS; then, the cells and PBS washing solution were collected into the same tube with the cultured supernatants. Thereafter, cells were centrifuged at 300× *g* for 5 min, washed with PBS, centrifuged at 300× *g* for 5 min again, and then stained with Annexin V-FITC for 15 min and propidium iodide (PI) for 5 min. The apoptotic cells were analyzed via flow cytometry (Navios, Beckman Coulter, Inc., Brea, CA, USA).

### 2.15. Western Blot

The extracted protein lysates from ischemic myocardial tissues and cardiomyocytes were used for Western blot analysis as described previously [27]. The lysates were normalized to equal amounts of protein, and 10 μg protein from cell lysates or 30 μg protein from tissue lysates were separated by 10% or 12% SDS–PAGE, transferred to polyvinylidene difluoride membrane, and then sealed with 5% nonfat milk in TBST for 3 h. Membranes were incubated with the following primary antibodies: p-AMPK (1:2000, #ab133448, Abcam), AMPK (1:1000, #ab80039, Abcam), PGC-1α (1:1000, #ab191838, Abcam), SIRT3 (1:1000, #ab264041, Abcam), SOD2 (1:5000, #ab13533, Abcam), Bax (1:2000, #ab182733, Abcam), B-cell-lymphoma protein 2 (Bcl-2) (1:2000, #ab182858, Abcam), caspase3 (1:2000, #ab184787, Abcam), cleaved-caspase3 (1:5000, #ab214430, Abcam), and β-tubulin (1:20,000, #66240-1-lg, Proteintech) at 4 °C overnight. Proteins were then probed with specific HRP-conjugated secondary antibodies for 30 min at room temperature. Finally, the protein bands were obtained using the ChemiDoc Touch Imaging System (Chemi Doc, Bio-Rad, Hercules, CA, USA), and the relative intensity of the bands was quantified using Image Lab software.

### 2.16. Molecular Docking Analysis

The molecular docking analysis between ICS II and SIRT3 was performed using Autodock 4.2 and Autodock Tools. The human X-ray crystal structure of SIRT3 (Protein Data Bank (PDB) ID: 3GLS) was obtained from the PDB archives and used as a target for molecular docking. To prepare compounds for the molecular docking, the 2D structure of ICS II was obtained from PubChem (RRID: SCR_004284) and then converted to a 3D structure. The molecular docking results were written as a pose viewer file, and the protein-ligand complex interactions were studied using the PyMOL molecular graphics system as described previously [28].

### 2.17. Statistical Analyses

Data are expressed as mean ± SEM and analyzed using GraphPad Prism 8.0.2 (GraphPad Software Inc., San Diego, CA, USA). Some data were normalized to control for unwanted sources of variation as follows: Firstly, the data were normalized to bring all of the variations into proportion with one another after deleting any outliers in various groups. Then, the coefficients associated with each variable were properly scaled to adjust for the disparity in the variable sizes. The cell viability of the control group was considered to be 100%, and the cell viability of ICS II treatment groups was displayed as a percentage of the control group. The PGC-1α, SIRT3, SOD2, Bax, and Bcl-2 expressions were normalized to that of β-tubulin, the phosphorylation level of AMPK was normalized to that of the total AMPK, and the activity level of caspase3 was normalized to that of the total caspase3. The protein expressions or levels of ICS II treatment groups are presented as fold changes in the sham or control group, the expression of which was set to 1. Two or multiple groups were compared using Student’s unpaired *t*-test or one-way ANOVA, followed by the Bonferroni post hoc test, with F at *p* < 0.05 and no significant variance inhomogeneity. *p* < 0.05 was considered statistically significant.

## 3. Results

### 3.1. ICS II Mitigated MI-Induced Myocardial Injury in Mice

**Figure 1:**
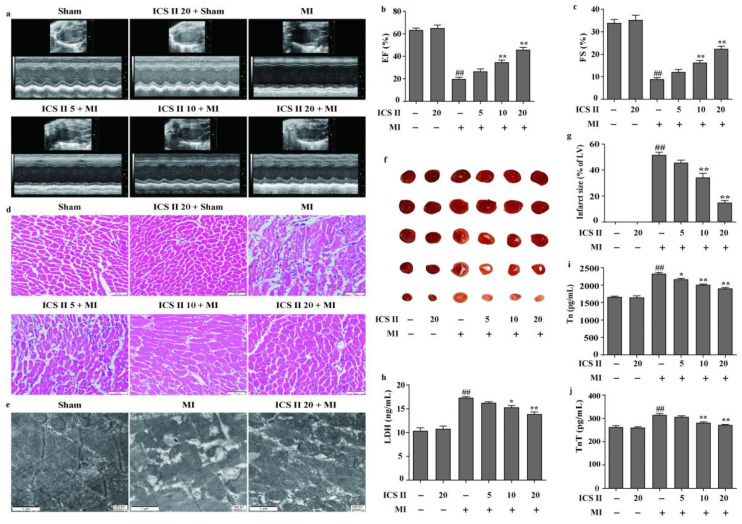
ICS II alleviated MI-induced myocardial damage in mice: (**a**) cardiac function was measured using Doppler echocardiography; (**b**) EF (%) (*n* = 6); (**c**) FS (%) (*n* = 6); (**d**) HE staining was performed to observe the pathological changes in the left ventricle. Magnification ×400; scale bar, 50 μm; (**e**) mitochondrial morphology was observed via TEM. Magnification ×30,000; scale bar, 1 μm; (**f**) myocardial infarct size was performed via TTC staining; (**g**) quantitative data of myocardial infarct size (%) (*n* = 5); (**h**) LDH activity (*n* = 6); (**i**) Tn level (*n* = 6); (**j**) TnT level (*n* = 6). The data are presented as the mean ± SEM. ^##^ *p* < 0.01 vs. sham; * *p* < 0.05, ** *p* < 0.01 vs. MI.

The left anterior descending coronary artery ligation-induced MI mice model was used to determine the effect of ICS II on MI-induced myocardial injury. The results showed that MI reduced left ventricular EF and FS in mice than those of the sham group; however, ICS II improved cardiac function, as reflected by enhanced EF and FS after MI insult (Figure 1a–c). Next, the effect of ICS II on pathological changes and mitochondrial ultrastructure of the left ventricle were assessed via HE staining and TEM, respectively. The results demonstrated that myocardial hypertrophy and inflammatory infiltration were obviously observed in the MI group in contrast to those of the sham group in mice (Figure 1d). Moreover, MI resulted in mitochondrial vacuolization and a reduction in matrix density in comparison with the sham group, while ICS II significantly reversed these changes (Figure 1e). Furthermore, the results indicated that myocardial infarct size was obviously increased in the MI group compared with that of the sham group in mice. However, ICS II markedly decreased the myocardial infarct size compared with the MI group, as evidenced by TTC staining in mice (Figure 1f,g). Additionally, ICS II also markedly decreased the levels of LDH, Tn, and TnT in serum compared with the MI group in mice (Figure 1h–j). These findings indicate that ICS II effectively mitigates MI-induced myocardial damage in mice.

### 3.2. Microarray Data Analyses

**Figure 2:**
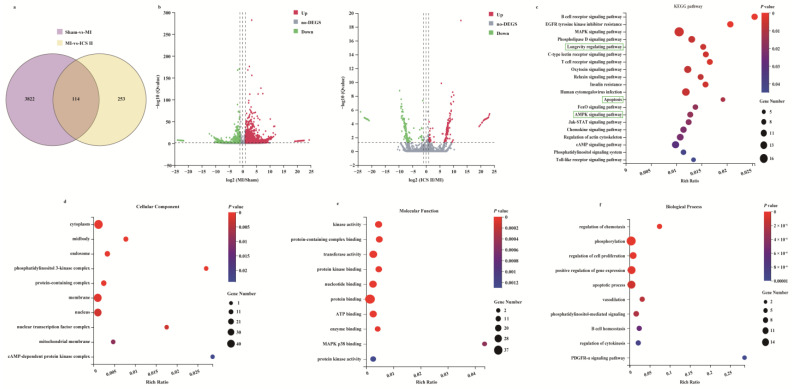
Analysis of DEGs profiling: (**a**) the DEGs between sham versus MI groups and ICS II versus MI groups were summarized with a Venn diagram; (**b**) the DEGs between MI versus sham groups and ICS II versus MI groups were summarized with Volcano maps; (**c**) KEGG pathways enriched with DEGs and their matching *p*-values; (**d**–**f**) dot plot showed the fold enrichment values for the genes, which were analyzed by significant GO terms using *p*-values.

To systemically explore the mechanism of ICS II on MI, the RNAseq data of ischemic myocardial samples were analyzed. The results showed that 3936 DEGs were identified in the MI group compared with those of the sham group, while 367 DEGs were identified in ICS II versus MI groups (Figure 2a,b). Furthermore, 114 out of the 367 DEGs responding to the ICS II treatment were associated with DEGs elicited by MI injury, as evidenced by the Venn diagram (Figure 2a). Moreover, the KEGG pathway and enrichment of GO terms for the selected 367 DEGs involved were studied. The KEGG pathway analysis indicated that AMPK and apoptosis signaling pathways were significantly enriched (Figure 2c). Moreover, enrichment analyses for cellular components, molecular function, and biological process showed that kinase activity, protein kinase binding, and apoptotic process were evidently enriched (Figure 2d–f). These results demonstrate that the anti-MI effect of ICS II might be related to the regulation of AMPK and apoptosis signaling pathways.

### 3.3. ICS II Alleviated Oxidative Stress and Activated AMPK/PGC-1α/SIRT3 Signaling Pathway after MI in Mice

**Figure 3:**
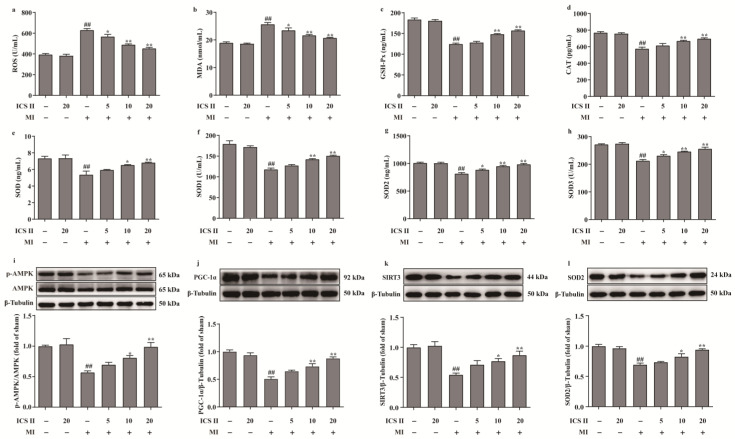
ICS II alleviated oxidative stress injury and activated AMPK/PGC-1α/SIRT3 signaling pathway in MI-induced mice: (**a**) ROS level (*n* = 6); (**b**) MDA level (*n* = 6); (**c**) GSH-Px activity (*n* = 6); (**d**) CAT activity (*n* = 6); (**e**) SOD activity (*n* = 6); (**f**) SOD1 activity (*n* = 6); (**g**) SOD2 activity (*n* = 6); (**h**) SOD3 activity (*n* = 6); (**i**) expression and quantitation of p-AMPK (*n* = 5); (**j**) expression and quantitation of PGC-1α (*n* = 5); (**k**) expression and quantitation of SIRT3 (*n* = 5); (**l**) expression and quantitation of SOD2 (*n* = 5). The data are presented as the mean ± SEM. ^##^ *p* < 0.01 vs. sham; * *p* < 0.05, ** *p* < 0.01 vs. MI.

The levels of ROS and MDA, and the activities of CAT, GSH-Px, SOD, SOD1, SOD2, and SOD3 were determined to elucidate the effect of ICS II on oxidative stress after MI insult. The results showed that the levels of ROS and MDA were elevated, and the activities of CAT, GSH-Px, SOD, SOD1, SOD2, and SOD3 were decreased after MI in mice, whereas ICS II evidently reversed these changes (Figure 3a–h). These findings demonstrated that ICS II protected against MI-induced evident oxidative stress. Furthermore, the phosphorylation level of AMPK and the protein expressions of PGC-1α, SIRT3, and SOD2 were significantly downregulated in mice of the MI group, while ICS II obviously reversed these changes (Figure 3i–l). The above findings imply that ICS II ameliorates MI-induced oxidative stress by activating the AMPK/PGC-1α/SIRT3 signaling pathway.

### 3.4. ICS II Inhibited Myocardial Apoptosis in Mice after MI

**Figure 4:**
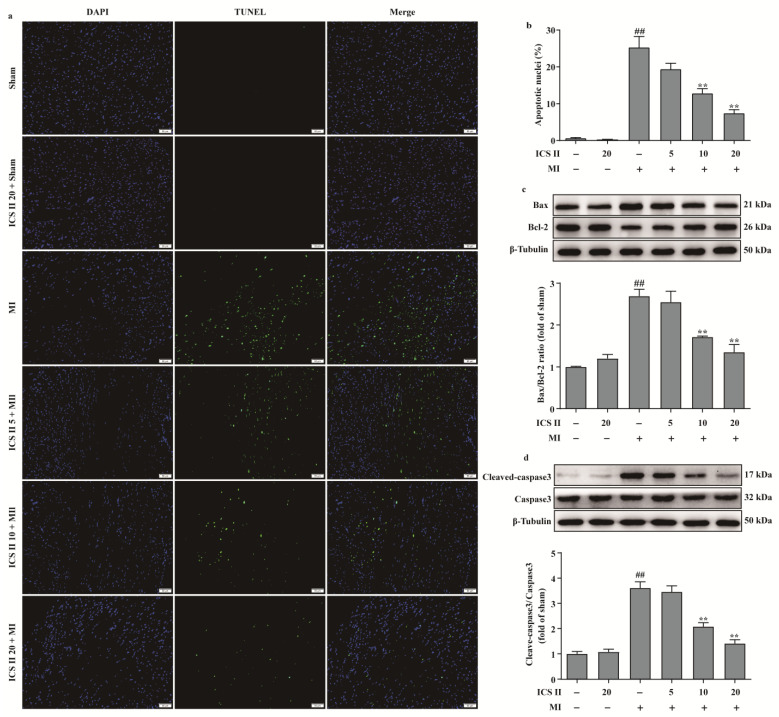
ICS II inhibited myocardial apoptosis in MI-induced mice: (**a**) myocardial apoptosis was assessed using TUNEL staining. Magnification ×200; scale bar, 50 μm; (**b**) quantitation of TUNEL-positive nuclei (%) in the left ventricle (*n* = 5); (**c**) expression of Bax and Bcl-2 and quantitation of Bax/Bcl-2 ratio (*n* = 5); (**d**) cleaved-caspase3 level and quantitation (*n* = 5). The data are presented as the mean ± SEM. ^##^ *p* < 0.01 vs. sham; ** *p* < 0.01 vs. MI.

We next investigated the role of apoptosis in the inhibitory effect of ICS II on MI. The results showed that ICS II reduced MI-induced myocardial apoptosis, as evidenced by a decreased number of TUNEL-positive nuclei (Figure 4a,b). In addition, the ratio of Bax/Bcl-2 and the level of cleaved-caspase3 were significantly increased after MI; however, ICS II decreased the ratio of Bax/Bcl-2 and the level of cleaved-caspase3 in mice compared with the MI group (Figure 4c,d). Thus, these findings suggest that ICS II reduces MI-induced myocardial apoptosis in mice.

### 3.5. ICS II Protected against OGD-Induced Cardiomyocyte Injury

**Figure 5:**
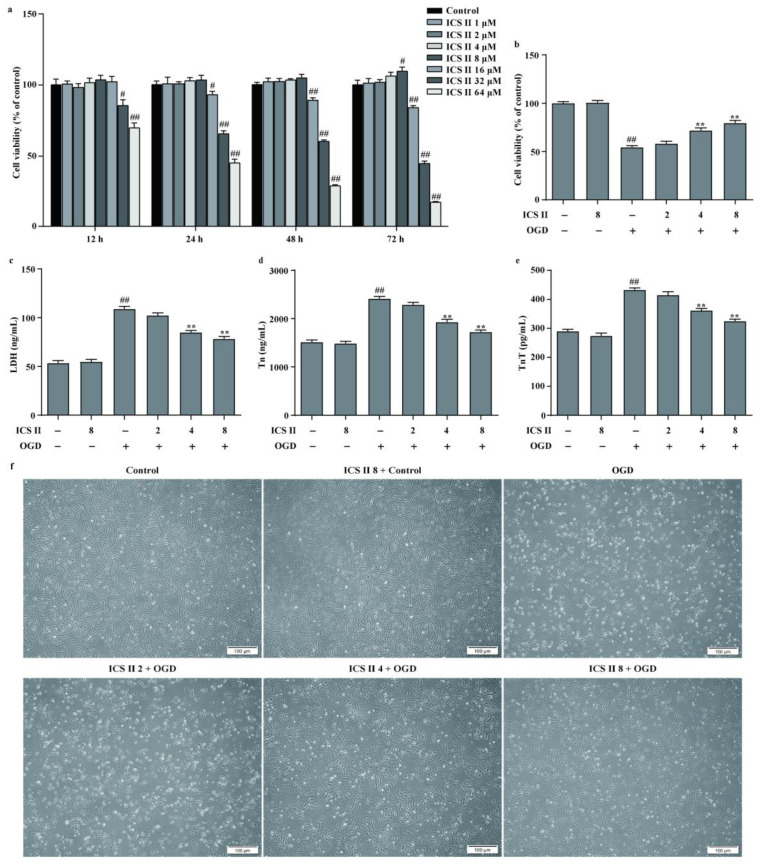
ICS II protected against OGD-induced cardiomyocyte injury: (**a**) effect of ICS II on cardiomyocytes (*n* = 6); (**b**) cell viability was detected via the MTT assay (*n* = 6); (**c**) cytotoxicity was determined using the LDH assay (*n* = 6); (**d**) Tn level (*n* = 6); (**e**) TnT level (*n* = 6); (**f**) the morphology of cardiomyocytes was observed using a reverse-phase microscope. Magnification ×100; scale bar, 100 μm. ^#^ *p* < 0.05, ^##^ *p* < 0.01 vs. control; ** *p* < 0.01 vs. OGD.

To further confirm the role of ICS II during MI, the OGD model in H9c2 cardiomyocytes was applied to mimic the MI model. The results showed that ICS II exerted no effect on cardiomyocytes below 8 μM within 48 h (Figure 5a). Furthermore, ICS II significantly increased cell viability and reduced the amount of LDH released upon OGD, as reflected by MTT and LDH assays (Figure 5b,c). Additionally, Tn and TnT levels were increased, whereas cardiomyocytes were shrunk, depleted in cell numbers, and even died after OGD insult. However, ICS II apparently reversed these changes (Figure 5d–f). These findings confirm that ICS II effectively protects against OGD-induced cardiomyocyte injury.

### 3.6. ICS II Suppressed OGD-Induced Mitochondrial Oxidative Stress by Activating AMPK/PGC-1α/SIRT3 Signaling Pathway

**Figure 6:**
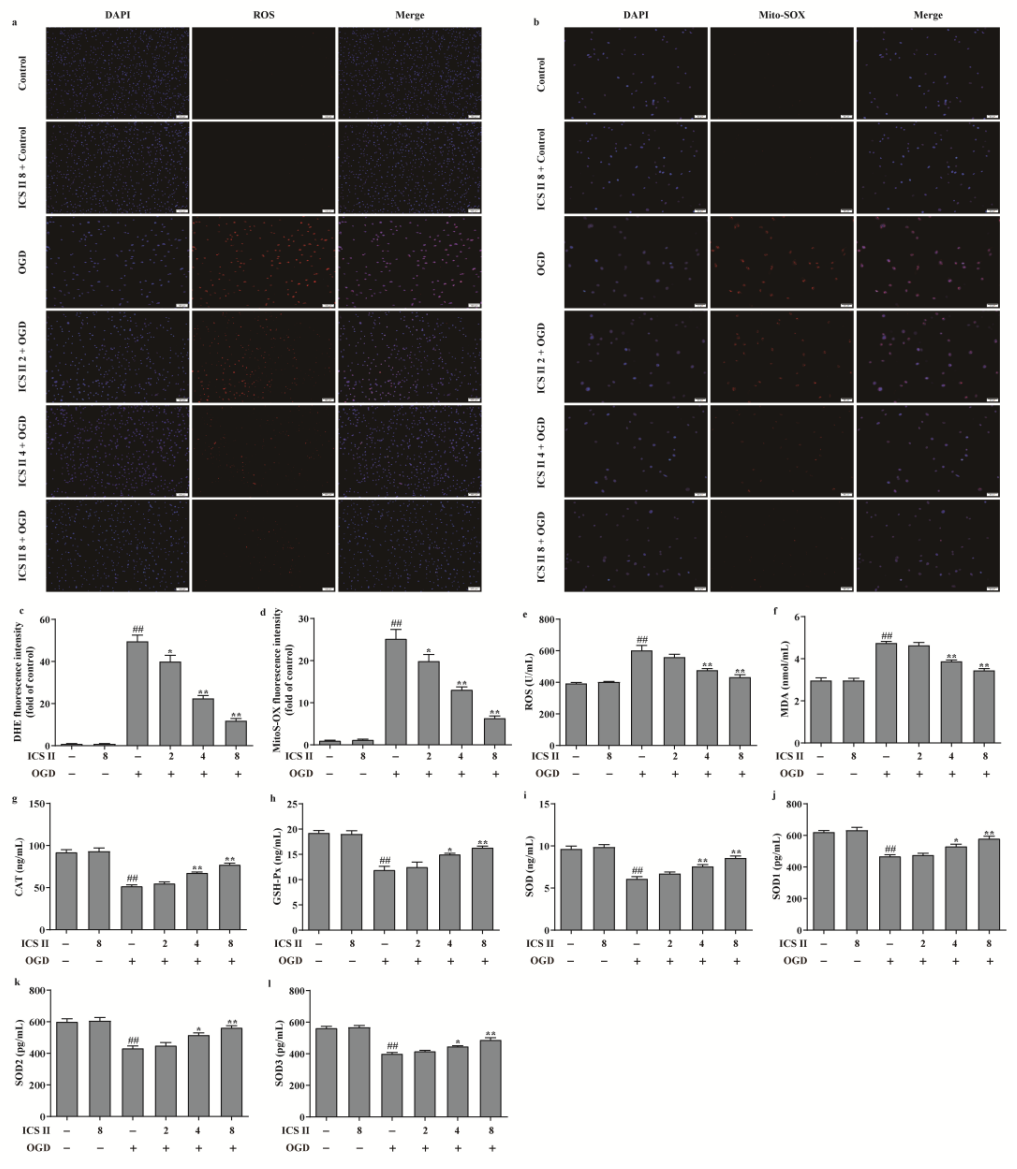
ICS II suppressed OGD-induced intracellular and mitochondrial oxidative stress: (**a**) DHE staining. Magnification ×100; scale bar, 100 μm; (**b**) Mito-SOX staining. Magnification ×100; scale bar, 50 μm; (**c**) quantitative analyses of DHE fluorescence intensity (*n* = 5); (**d**) quantitative analyses of Mito-SOX fluorescence intensity (*n* = 5); (**e**) ROS level (*n* = 6); (**f**) MDA level (*n* = 6); (**g**) CAT activity (*n* = 6); (**h**) GSH-Px activity (*n* = 6); (**i**) SOD activity (*n* = 6); (**j**) SOD1 activity (*n* = 6); (**k**) SOD2 activity (*n* = 6); (**l**) SOD3 activity (*n* = 6). The data are presented as the mean ± SEM. ^##^ *p* < 0.01 vs. control; * *p* < 0.05, ** *p* < 0.01 vs. OGD.

**Figure 7:**
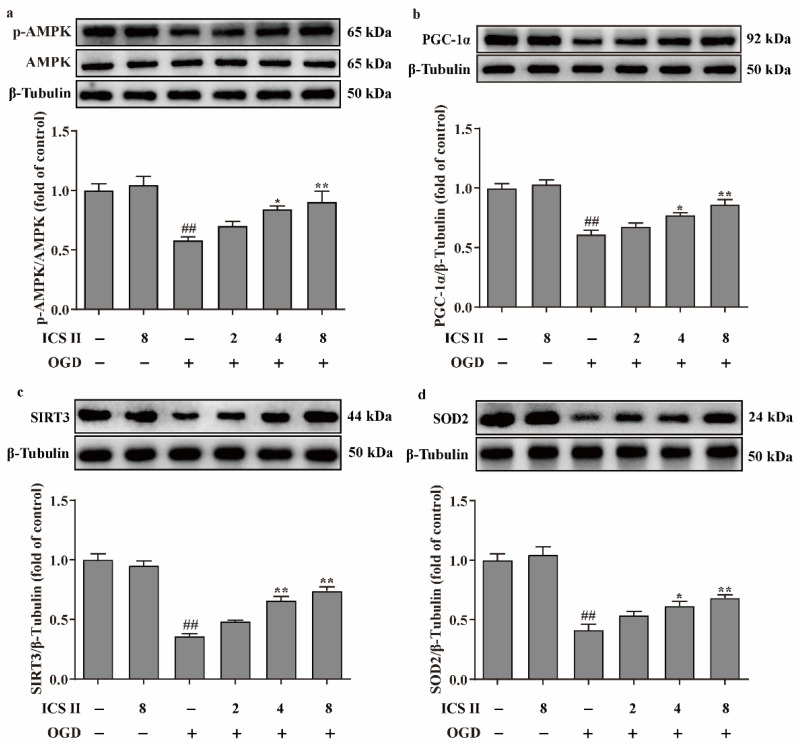
ICS II activated AMPK/PGC-1α/SIRT3 signaling pathway in OGD-induced cardiomyocytes injury: (**a**) Expression and quantitation of p-AMPK (*n* = 5); (**b**) expression and quantitation of PGC-1α (*n* = 5); (**c**) expression and quantitation of SIRT3 (*n* = 5); (**d**) expression and quantitation of SOD2 (*n* = 5). The data are presented as the mean ± SEM. ^##^ *p* < 0.01 vs. control; * *p* < 0.05, ** *p* < 0.01 vs. OGD.

To further verify the effect of ICS II on OGD-induced oxidative stress, intracellular ROS and mitochondrial O_2_^•^^−^ generation were evaluated via DHE staining and Mito-SOX staining, respectively. The results showed that the red fluorescence was enhanced after OGD in comparison with the control group, which indicated that OGD accelerated the generation of intracellular ROS and mitochondrial O_2_^•^^−^. However, ICS II significantly decreased intracellular ROS and mitochondrial O_2_^•^^−^ generation, as evidenced by the weakened red fluorescence intensity (Figure 6a–d). Furthermore, the levels of ROS and MDA were also elevated, and the activities of CAT, GSH-Px, SOD, SOD1, SOD2, and SOD3 were suppressed after OGD insult; by contrast, ICS II markedly reversed these changes (Figure 6e–l). In keeping with the results in vivo, the phosphorylation level of AMPK and the protein expressions of PGC-1α, SIRT3, and SOD2 were decreased after OGD insult, while ICS II obviously reversed these changes (Figure 7a–d). The aforementioned results illustrate that ICS II ameliorates OGD-induced mitochondrial oxidative stress by activating the AMPK/PGC-1α/SIRT3 signaling pathway.

### 3.7. ICS II Reduced OGD-Induced Cardiomyocytes Apoptosis

**Figure 8:**
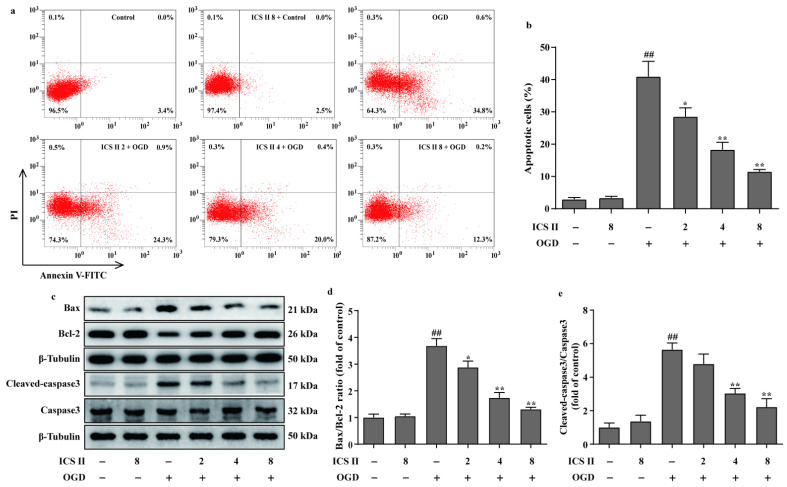
ICS II reduced OGD-induced cardiomyocyte apoptosis: (**a**) cardiomyocyte apoptosis was performed via flow cytometry; (**b**) quantitation of apoptotic percentage (%) (*n* = 4); (**c**) representative Western blots of Bax and Bcl-2 expressions and representative Western blots of caspase3 and cleaved-caspase3 levels; (**d**) quantitation of Bax/Bcl-2 ratio (*n* = 5); (**e**) quantitation of cleaved-caspase3/caspase3 ratio (*n* = 5). The data are presented as the mean ± SEM. ^##^ *p* < 0.01 vs. control; * *p* < 0.05, ** *p* < 0.01 vs. OGD.

The effect of ICS II on cardiomyocyte apoptosis was subsequently explored using flow cytometry. The results showed that apoptotic cardiomyocytes were obviously elevated after OGD; however, ICS II reduced OGD-induced cardiomyocyte apoptosis, as evidenced by the reduced number of apoptotic cells (Figure 8a,b). In addition, ICS II downregulated the ratio of Bax/Bcl-2 and the level of cleaved-caspase3 in OGD-induced cardiomyocytes, consistent with the in vivo findings (Figure 8c–e).

### 3.8. Prediction of Therapeutic Targets of ICS II against MI

**Figure 9:**
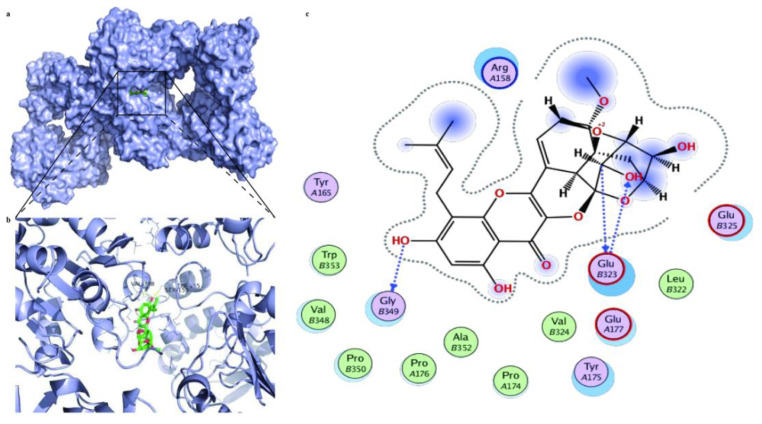
The molecular docking results of ICS II with the SIRT3 complex: (**a**) the whole view of the ICS II dimer and SIRT3 displaying the molecular binding pocket; (**b**) crystal structure of ICS II (green) displaying SIRT3 (blue) bound to the docking pocket; (**c**) residues of amino acids between ICS II with the SIRT3 complex.

Molecular docking was further utilized to predict the therapeutic targets of ICS II against MI. The results displayed a strong binding affinity between ICS II and SIRT3, with a binding energy of −6.25 kcal/mol. Furthermore, the presumptive binding modes and the pocket of amino acid sites including SER159, VAL348, and TYR165 further confirmed that ICS II bound to the hydrophobic pocket of SIRT3 (Figure 9). These findings demonstrate that SIRT3 may be a potential therapeutic target of ICS II against MI.

### 3.9. Effect of ICS II on SIRT3-KO Mice

**Figure 10:**
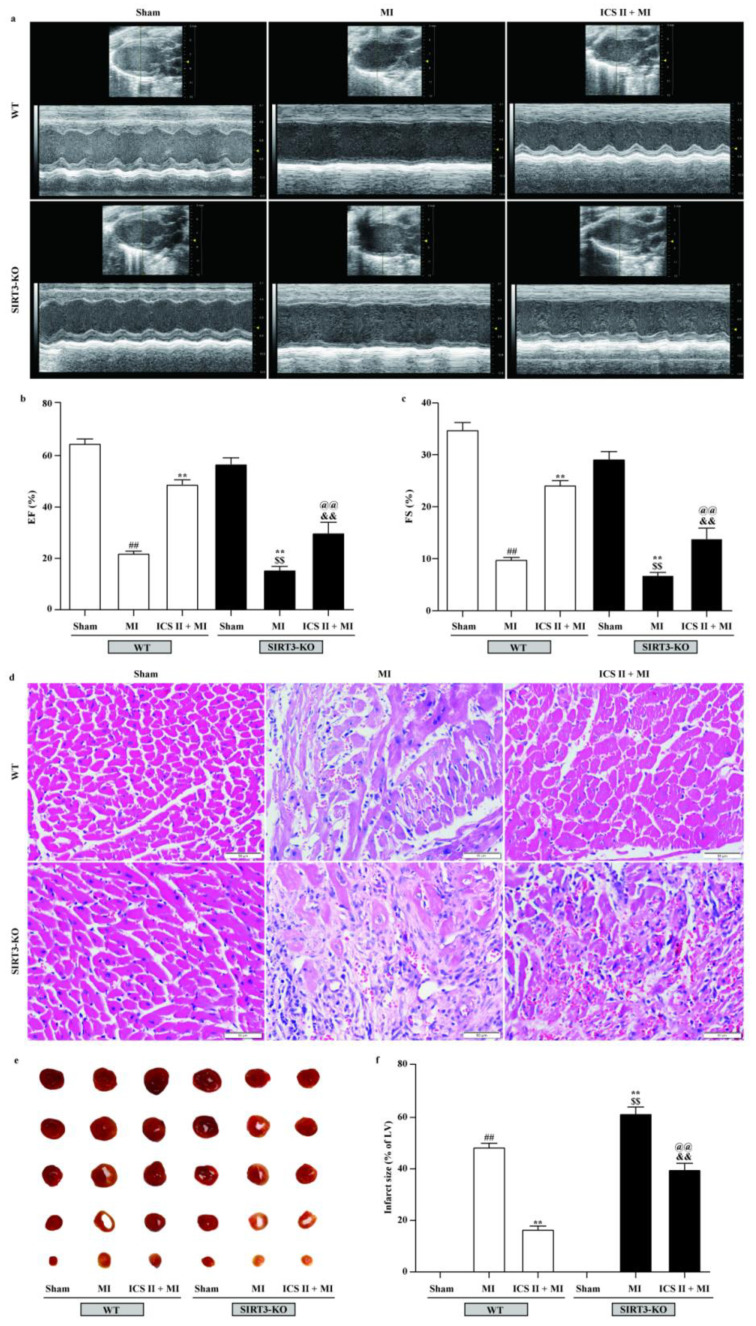
Effect of ICS II on SIRT3-KO mice: (**a**) cardiac function was measured using Doppler echocardiography; (**b**) EF (%) (*n* = 6); (**c**) FS (%) (*n* = 6); (**d**) HE staining was performed to observe the pathological changes in the left ventricle. Magnification ×400; scale bar, 50 μm; (**e**) myocardial infarct size was performed by TTC staining; (**f**) quantitative data of myocardial infarct size (%) (*n* = 5). The data are presented as the mean ± SEM. ^##^ *p* < 0.01 vs. sham (WT); ** *p* < 0.01 vs. MI (WT); ^$$^ *p* < 0.01 vs. sham (SIRT3-KO); ^@@^ *p* < 0.01 vs. MI (SIRT3-KO); ^&&^ *p* < 0.01 vs. ICS II + MI (WT).

To further verify if loss of functional SIRT3 offsets ICS II preconditioning-induced cardioprotection in MI, WT and SIRT3-KO mice were treated with or without ICS II for 7 days prior to MI. The results showed that SIRT3-KO mice exhibited more severe injury after MI insult than WT mice, as evidenced by their aggravated cardiac function and pathological changes, as well as augmented infarct size, while the absence of SIRT3 substantially abolished the protective effect of ICS II against MI (Figure 10). These results manifest that ICS II-mediated cardioprotection is, at least partly, dependent on the presence of SIRT3, and SIRT3 might be a potential target of ICS II.

## 4. Discussion

The current study revealed that (1) ICS II, a naturally occurring SIRT3 agonist derived from *Herbal Epimedii*, exerts cardioprotection against MI in vivo and in vitro; (2) the protective effects of ICS II on MI are due to a reduction in oxidative stress injury and apoptosis through activating the AMPK/PGC-1α/SIRT3 pathway; (3) SIRT3 is necessarily required for the development of cardioprotective effects, and SIRT3 deficiency largely abrogates the cardioprotective effects of ICS II.

MI is the most lethal manifestation of coronary heart disease, and exploring effective cardioprotective agents is extremely needed in the clinic. In the present study, we investigated the cardioprotective effects of ICS II, a natural, small molecule monomer, against MI injury and endeavored to expound its potential mechanisms. Our findings revealed that cardiac dysfunction and pathological changes were markedly observed, and the myocardial infarct size and LDH, Tn, and TnT levels were significantly increased after MI insult. These results indicated that the MI model was accepted according to a previous study [13]. However, ICS II effectively protected against MI damage, as indicated by an enhanced cardiac function, alleviated pathological changes, reduced myocardial infarct size, and decreased LDH, Tn, and TnT levels, suggesting that ICS II is a promising agent to translate into the clinic for preventing ischemic heart disease. Nevertheless, to clarify its precise mechanism, more detailed studies are required.

Notably, RNAseq analysis revealed that AMPK and apoptosis signaling pathways were significantly enriched by KEGG pathway analysis. AMPK has recently emerged as a redox sensor that plays an essential role in regulating oxidative stress and apoptosis. Since under oxidative stress, the accumulation of ROS negatively affects cardiac calcium handling, causes mitochondrial permeability transition pore opening, and contributes to cardiomyocyte apoptosis [29], hindering excessive oxidative stress and apoptosis may be a promising therapeutic strategy for MI. Thus, we hypothesized that the beneficial effect of ICS II might be related to the regulation of oxidative stress and apoptosis. As expected, our findings manifested MI-induced oxidative stress injury, thereby leading to mitochondrial dysfunction and apoptosis, in keeping with the theory of previous reports [30]; by contrast, ICS II effectively reduced the levels of oxidative stress biomarkers (ROS and MDA) and facilitated the antioxidant enzyme activities (CAT, GSH-Px, SOD, SOD1, SOD2, and SOD3) after MI insult. Moreover, ICS II also decreased the ratio of Bax/Bcl-2 and the level of cleaved-caspase3, which is the apoptotic executive protein after MI. The aforementioned findings manifested that ICS II protected against MI-induced myocardial injury, at least partially, by reducing oxidative stress and apoptosis. However, further mechanistic studies are needed to clarify this result. Notably, previous studies have demonstrated that the activation of the AMPK/PGC-1α/SIRT3 pathway reduces mitochondrial ROS overproduction and apoptosis by enhancing SOD2 activity [31,32]. Based on the above theories, we hypothesized that ICS II mediated redox homeostasis through activating the AMPK/PGC-1α/SIRT3 pathway. As we expected, the findings revealed that the phosphorylation level of AMPK and the expression of PGC-1α were downregulated after MI, in keeping with the results of a previous report [8], while ICS II increased the phosphorylation level of AMPK and the expression of PGC-1α, which confirmed that ICS II protected against MI, at least partly, through the activation of the AMPK/PGC-1α/SIRT3 pathway. Additionally, OGD-induced cardiomyocyte injury was used to emulate MI injury in vitro to further verify the mechanism of ICS II-mediated cardioprotective effects. As we expected, ICS II effectively improved cell viability, reduced cytotoxicity, and decreased the levels of Tn and TnT after OGD insult. Moreover, ICS II also decreased the intracellular ROS and mitochondrial O_2_^•^^−^ generation, elevated antioxidant enzyme activities, and reduced cardiomyocyte apoptosis after OGD. Most importantly, the AMPK phosphorylation and the activation of PGC-1α and SIRT3 were also promoted by ICS II after the OGD insult. These results further confirmed that ICS II effectively ameliorated OGD-induced oxidative stress and apoptosis through activating the AMPK/PGC-1α/SIRT3 pathway, consistent with the findings in vivo.

More interestingly, SIRT3 can increase mitochondrial energy production and boost the SOD2 activity, thereby reducing cardiac oxidative stress [33]. SOD2 is not only a key antioxidant enzyme to protect against superoxide production in the mitochondria but also the direct substrate of SIRT3, which is a mitochondrial nicotinamide adenine dinucleotide-dependent histone deacetylase [33]. Our results found that ICS II not only increased SIRT3 activity but also augmented SOD2 expression, thereby maintaining redox homeostasis. Thus, we hypothesized that SIRT3 might be a promising therapeutic target of ICS II against MI. Intriguingly, a direct interaction between ICS II and SIRT3 was revealed by the results of molecular docking, which preliminarily verified our hypothesis. Furthermore, ICS II was unable to protect against MI in SIRT3-KO mice, which indicated that ICS II-mediated cardioprotection mainly required the modulation of SIRT3.

Despite the encouraging experimental evidence, there are still limitations in the present study. Firstly, whether ICS II can develop a novel naturally occurring SIRT3 agonist still needs to be further probed even though we offered direct proof that ICS II could bind to SIRT3. Secondly, the detailed mechanism of ICS II against MI and the effect of post-treatment with ICS II on myocardial ischemia–reperfusion deserve further in-depth exploration.

## 5. Conclusions

Collectively, the present study illustrates that ICS II protects against MI-induced oxidative stress and apoptosis by targeting SIRT3 via activating the AMPK/PGC-1α signaling pathway. These findings raise the feasibility that ICS II might be a potent SIRT3 agonist against MI.

## Data Availability

All data related to this research are presented in the manuscript.

## References

[1] Ramachandra C.J.A., Hernandez-Resendiz S., Crespo-Avilan G.E., Lin Y.H., Hausenloy D.J. (2020). Mitochondria in acute myocardial infarction and cardioprotection. EBioMedicine.

[2] Anderson J.L., Morrow D.A. (2017). Acute Myocardial Infarction. N. Engl. J. Med..

[3] Marin-Juez R., El-Sammak H., Helker C.S.M., Kamezaki A., Mullapuli S.T., Bibli S.I., Foglia M.J., Fleming I., Poss K.D., Stainier D.Y.R. (2019). Coronary Revascularization during Heart Regeneration Is Regulated by Epicardial and Endocardial Cues and Forms a Scaffold for Cardiomyocyte Repopulation. Dev. Cell.

[4] Rentrop K.P., Feit F. (2015). Reperfusion therapy for acute myocardial infarction: Concepts and controversies from inception to acceptance. Am. Heart J..

[5] Bonaca M.P., Sabatine M.S. (2016). Antiplatelet Therapy for Long-Term Secondary Prevention after Myocardial Infarction. JAMA Cardiol..

[6] Li Y., Yu H., Zhao L., Zhu Y., Bai R., Jin Z., Fu Z., Zhang X., Su J., Liu H. (2020). Effects of carbon nanotube-mediated Caspase3 gene silencing on cardiomyocyte apoptosis and cardiac function during early acute myocardial infarction. Nanoscale.

[7] Zhang Q., Wang L., Wang S., Cheng H., Xu L., Pei G., Wang Y., Fu C., Jiang Y., He C. (2022). Signaling pathways and targeted therapy for myocardial infarction. Signal Transduct. Target Ther..

[8] Zhang L., Wang Y.N., Ju J.M., Shabanova A., Li Y., Fang R.N., Sun J.B., Guo Y.Y., Jin T.Z., Liu Y.Y. (2021). Mzb1 protects against myocardial infarction injury in mice via modulating mitochondrial function and alleviating inflammation. Acta Pharmacol. Sin..

[9] Wallert M., Ziegler M., Wang X., Maluenda A., Xu X., Yap M.L., Witt R., Giles C., Kluge S., Hortmann M. (2019). alpha-Tocopherol preserves cardiac function by reducing oxidative stress and inflammation in ischemia/reperfusion injury. Redox Biol..

[10] Kibel A., Lukinac A.M., Dambic V., Juric I., Selthofer-Relatic K. (2020). Oxidative Stress in Ischemic Heart Disease. Oxidative Med. Cell. Longev..

[11] Redza-Dutordoir M., Averill-Bates D.A. (2016). Activation of apoptosis signalling pathways by reactive oxygen species. Biochim. Biophys. Acta.

[12] Teringova E., Tousek P. (2017). Apoptosis in ischemic heart disease. J. Transl. Med..

[13] Yu Y., Liu H., Yang D., He F., Yuan Y., Guo J., Hu J., Yu J., Yan X., Wang S. (2019). Aloe-emodin attenuates myocardial infarction and apoptosis via up-regulating miR-133 expression. Pharmacol. Res..

[14] Ye L., Li M., Wang Z., Yang Z., Zhang J., Fang H., He Z., Wang X. (2020). Depression of Mitochondrial Function in the Rat Skeletal Muscle Model of Myofascial Pain Syndrome Is through Down-Regulation of the AMPK-PGC-1alpha-SIRT3 Axis. J. Pain Res..

[15] Marino A., Hausenloy D.J., Andreadou I., Horman S., Bertrand L., Beauloye C. (2021). AMP-activated protein kinase: A remarkable contributor to preserve a healthy heart against ROS injury. Free Radic. Biol. Med..

[16] Kukidome D., Nishikawa T., Sonoda K., Imoto K., Fujisawa K., Yano M., Motoshima H., Taguchi T., Matsumura T., Araki E. (2006). Activation of AMP-activated protein kinase reduces hyperglycemia-induced mitochondrial reactive oxygen species production and promotes mitochondrial biogenesis in human umbilical vein endothelial cells. Diabetes.

[17] Zhang J., Xiang H., Liu J., Chen Y., He R.R., Liu B. (2020). Mitochondrial Sirtuin 3: New emerging biological function and therapeutic target. Theranostics.

[18] Fu S., Li Y.L., Wu Y.T., Yue Y., Qian Z.Q., Yang D.L. (2018). Icariside II attenuates myocardial fibrosis by inhibiting nuclear factor-kappaB and the TGF-beta1/Smad2 signalling pathway in spontaneously hypertensive rats. Biomed. Pharmacother..

[19] Sun Y.S., Thakur K., Hu F., Zhang J.G., Wei Z.J. (2020). Icariside II inhibits tumorigenesis via inhibiting AKT/Cyclin E/CDK 2 pathway and activating mitochondria-dependent pathway. Pharmacol. Res..

[20] Zheng Y., Deng Y., Gao J.M., Lv C., Lang L.H., Shi J.S., Yu C.Y., Gong Q.H. (2020). Icariside II inhibits lipopolysaccharide-induced inflammation and amyloid production in rat astrocytes by regulating IKK/IkappaB/NF-kappaB/BACE1 signaling pathway. Acta Pharmacol. Sin..

[21] Liu M.B., Wang W., Gao J.M., Li F., Shi J.S., Gong Q.H. (2020). Icariside II attenuates cerebral ischemia/reperfusion-induced blood-brain barrier dysfunction in rats via regulating the balance of MMP9/TIMP1. Acta Pharmacol. Sin..

[22] Feng L., Gao J., Liu Y., Shi J., Gong Q. (2018). Icariside II alleviates oxygen-glucose deprivation and reoxygenation-induced PC12 cell oxidative injury by activating Nrf2/SIRT3 signaling pathway. Biomed. Pharmacother..

[23] Wu Y., Qian Z., Fu S., Yue Y., Li Y., Sun R., Huang B., Yang D. (2018). IcarisideII improves left ventricular remodeling in spontaneously hypertensive rats by inhibiting the ASK1-JNK/p38 signaling pathway. Eur. J. Pharmacol..

[24] Guan B.F., Dai X.F., Huang Q.B., Zhao D., Shi J.L., Chen C., Zhu Y., Ai F. (2020). Icariside II ameliorates myocardial ischemia and reperfusion injury by attenuating inflammation and apoptosis through the regulation of the PI3K/AKT signaling pathway. Mol. Med. Rep..

[25] Gao E., Lei Y.H., Shang X., Huang Z.M., Zuo L., Boucher M., Fan Q., Chuprun J.K., Ma X.L., Koch W.J. (2010). A novel and efficient model of coronary artery ligation and myocardial infarction in the mouse. Circ. Res..

[26] Gao J., Long L., Xu F., Feng L., Liu Y., Shi J., Gong Q. (2020). Icariside II, a phosphodiesterase 5 inhibitor, attenuates cerebral ischaemia/reperfusion injury by inhibiting glycogen synthase kinase-3beta-mediated activation of autophagy. Br. J. Pharmacol..

[27] Ma L.L., Ding Z.W., Yin P.P., Wu J., Hu K., Sun A.J., Zou Y.Z., Ge J.B. (2021). Hypertrophic preconditioning cardioprotection after myocardial ischaemia/reperfusion injury involves ALDH2-dependent metabolism modulation. Redox Biol..

[28] Gao J., Chen N., Li N., Xu F., Wang W., Lei Y., Shi J., Gong Q. (2020). Neuroprotective Effects of Trilobatin, a Novel Naturally Occurring Sirt3 Agonist from Lithocarpus polystachyus Rehd., Mitigate Cerebral Ischemia/Reperfusion Injury: Involvement of TLR4/NF-kappaB and Nrf2/Keap-1 Signaling. Antioxid. Redox Signal.

[29] Peoples J.N., Saraf A., Ghazal N., Pham T.T., Kwong J.Q. (2019). Mitochondrial dysfunction and oxidative stress in heart disease. Exp. Mol. Med..

[30] Muntean D.M., Sturza A., Danila M.D., Borza C., Duicu O.M., Mornos C. (2016). The Role of Mitochondrial Reactive Oxygen Species in Cardiovascular Injury and Protective Strategies. Oxidative Med. Cell. Longev..

[31] Han L., Li J., Li J., Pan C., Xiao Y., Lan X., Wang M. (2020). Activation of AMPK/Sirt3 pathway by phloretin reduces mitochondrial ROS in vascular endothelium by increasing the activity of MnSOD via deacetylation. Food Funct..

[32] Liu M., Li X., Huang D. (2020). Mfn2 Overexpression Attenuates Cardio-Cerebrovascular Ischemia-Reperfusion Injury Through Mitochondrial Fusion and Activation of the AMPK/Sirt3 Signaling. Front. Cell Dev. Biol..

[33] Caballero E.P., Mariz-Ponte N., Rigazio C.S., Santamaria M.H., Corral R.S. (2020). Honokiol attenuates oxidative stress-dependent heart dysfunction in chronic Chagas disease by targeting AMPK/NFE2L2/SIRT3 signaling pathway. Free Radic. Biol. Med..

